# Traffic accident mortality change and its relation to ambient temperature in a dry region of Iran; a study using generalized additive mixed models

**Published:** 2019-08

**Authors:** Omid Aboubakri, Narges Khanjani, Younes Jahani, Bahram Bakhtiari

**Affiliations:** ^*a*^Neurology Research Center, Kerman University of Medical Sciences, Kerman, Iran.; ^*b*^Environmental Health Engineering Research Center, Kerman University of Medical Sciences, Kerman, Iran.; ^*c*^Department of Biostatistics and Epidemiology, School of Public Health, Kerman University of Medical Sciences, Kerman, Iran.; ^*d*^Water Engineering Department, Faculty of Agriculture, Shahid Bahonar University of Kerman, Kerman, Iran.

## Abstract

**Background:**

Traffic accidents is one of the main causes of mortality, and has a high impact on overall health and development. Iran is a country with a high prevalence of traffic accidents, and this has been a big concern. In this study, we aimed to study the past decade’s trend in mortality due to traffic accident in Kerman, Iran, and its relation with ambient temperature by using Generalized Additive Mixed Models.

**Methods:**

Meteorological data, such as temperature, humidity, and wind speed were obtained from the Meteorological Organization of Kerman and traffic mortality data were obtained from the Health Deputy of Kerman University of Medical Sciences. In order to explore the relations, Generalized Additive Mixed Models (GAMMs) were used. Additive models are nonparametric forms of regression models in which the summation of regression coefficients × explanatory variables of a linear regression is replaced by a summation of arbitrary smooth functions of the explanatory variables.

**Results:**

About 2900 deaths due to traffic accident occurred from March 2005 until May 2017 in Kerman. The monthly average deaths were 20.01(±14.43) that was more for men than women (15.42 vs 4.58), and was highest in the age 31-60 group. Non-linear effect of time on mortality was significant. There was a negative effect of time on mortality until 2009, while all other confounders were adjusted. After a short-term increasing trend till early 2011, and then a decreasing trend until 2013, an increasing trend was observed again. Holding pollutants, temperature and year (trend effect) stable, mortality tended to be higher for the first half of year, with an increasing slope in the first 3 months of the year. Monthly average temperatures below 13°C was related with increased mortality, but average temperatures around 20°C had no effect on mortality. High temperatures (>23°C) were associated with less mortality.

**Conclusions:**

Most traffic accident related deaths occurred in first half of year (spring and summer), and it is probably because of increased inter-city traveling during the summer break. Our results showed that high extreme temperatures have less contribution on the mortality.

**Keywords:**

Accident, Mortality, Trend, Ambient Temperature

